# The prevalence of domestic violence and its association with family factors: a cross-sectional study among pregnant women in urban communities of Hengyang City, China

**DOI:** 10.1186/s12889-020-08683-9

**Published:** 2020-05-05

**Authors:** Baohua Zheng, Xidi Zhu, Zhao Hu, Wensu Zhou, Yunhan Yu, Shilin Yin, Huilan Xu

**Affiliations:** Department of Social Medicine and Health Management, Xiangya School of Public Health, Central South University, Changsha, China

**Keywords:** Domestic violence (DV), Women in the third trimester of pregnancy, Family factors, China

## Abstract

**Background:**

With the increased vulnerability during pregnancy, domestic violence (DV) is a serious threat to the physical and mental health of pregnant women, making it a significant issue in public health initiatives. In China, family is of great significance to pregnant women, but few scholars have focused specifically on the relationship between the family factors of pregnant women and DV. This study aimed to explore the prevalence and association between family factors and DV among women in late pregnancy, to provide evidence for the prevention of domestic violence during pregnancy.

**Methods:**

A cross-sectional survey was conducted from July–October, 2019 among pregnant women in urban communities of Hengyang City, Hunan Province, China. A total of 813 participants were included by a multi-staged cluster random sampling method. DV was assessed by the Abuse Assessment Screen Questionnaire (AAS). A multivariate binary logistic regression model was used to evaluate the relationship between family factors and DV.

**Results:**

Ultimately, 127 (15.62%) participants were identified as victims of DV. After adjustment, the potential risk factors of DV were tensions between their mother-in-law and other family members (OR: 2.85; 95% CI: 1.29 to 6.30 and OR: 3.30; 95% CI: 1.57 to 6.93), medium household debt (OR: 2.17; 95% CI: 1.18 to 4.00), middle and low family APGARI (OR: 2.01; 95% CI: 1.30 to 3.13 and OR: 4.01; 95% CI: 2.09 to 7.69).

**Conclusions:**

In summary, women in late pregnancy were at higher risk of DV in the family with tensions, medium household debt and family dysfunction, which may help medical personnel intervene in cases of domestic violence against pregnant women in a reasonable and timely manner.

## Background

Domestic violence (DV) refers to all forms of violence against family members by means of beating, binding, maiming, abuse, restriction of personal freedom, abandonment and sexual abuse [1, 2]. DV is not only a serious social problem but also a public health problem, and has been reported in both developed and developing countries [3, 4]. Pregnancy is considered as a special period that DV starts or exacerbates [5, 6]. Some researchers found that pregnant women were 2.7 to 3.9 times more likely to suffer from physical violence and twice as likely to suffer from sexual violence compared with women who are not pregnant [7]. What’s more, because of the increased vulnerability women experience during pregnancy, DV not only will do harm to physical and mental health (disability, depression, substance abuse, etc.) [8, 9], but also may cause adverse pregnancy outcomes (premature birth, abortion, etc.), which seriously affect the health of the next generation [10].

Due to the different national cultures and the tools of assessment, studies conducted in pregnant women have reported that the prevalence of DV varies from 0.9 to 20% [11]. The prevalence of DV among pregnant women is even higher in some developing countries or underdeveloped regions, the reported prevalence of DV was 28.2% in Iran [12], 51% in Pakistan [13], 45.1% in Peru [14], 37% in Kenya [15]. DV include physical, mental and sexual violence. Physical violence was the second leading cause of trauma during pregnancy [16], and the prevalence varied from 0.9 to 21% worldwide [17–19]. A review including 18 studies from 1996 to 2009 reported that the prevalence of mental violence during pregnancy was 1.5 to 36% and that of sexual violence was 1.0 to 3.9% [20].

In previous studies, many factors were associated with the occurrence of DV among pregnant women. First, social instability was the potential risk factor associated with violence. For instance, some studies showed that compared to pregnant women who lived with their partners, had stable marriages and income, higher risk of DV tend to be reported among low-income, separated and divorced pregnant women [21–23]. Additionally, violence during pregnancy has been associated with some pregnancy characteristics, such as multiple deliveries [24], lack of prenatal care [25] and unintended pregnancy [5, 21]. Moreover, there has been some indication that violence during pregnancy is associated with unhealthy lifestyle behaviours, such as drinking, smoking, unhealthy diet and substance abuse [26]. Some characteristics of pregnant women’s intimate partners, such as alcohol misuse, jealousy, stress [26], and unemployment [27, 28] might be risk factors of DV during pregnancy. Based on the current literature, we found that the relationship between family factors and DV among pregnant women has not received much attention. Few studies have explored the relationship between family characteristics and DV from the various perspectives of family characteristics in China. Therefore, this study focuses on the association between DV among pregnant women and four family characteristics: family relationships, family structure, family-related stressors and family function, to facilitate DV intervention for pregnant women.

## Methods

### Research design and sample

A cross-sectional survey was conducted from July–October, 2019 among pregnant women in urban communities of Hengyang City, Hunan Province, China. A multi-staged cluster random sampling method was used in this study. There are 5 districts in urban Hengyang. In the first stage, a street from each district was randomly selected: Zhengxiang Street, Qingshan Street, Baishazhou Street, Guangdonglu Street and Zhurong Street. In the second stage, proportional sampling was carried out at a proportion of 1/3 to randomly select 4 communities in Zhengxiang Street, 3 communities in Qingshan Street, 3 communities in Baishazhou Street, 2 communities in Guangdonglu Street, and 2 communities in Zhurong Street. In total, 14 communities were selected for this study. All pregnant women who were registered in community health service centres and who met the inclusion criteria were potential subjects in this study. The inclusion criteria for the study were as follows: 1. women in the third trimester of pregnancy; 2. pregnant women over 16 years old; and 3. pregnant women who had local household registration, or migrant people who had lived in urban areas of Hengyang city for more than 6 months. The exclusion criteria were: 1. pregnant women with cognitive disorders, severe mental illnesses or other serious diseases who cannot fill out the questionnaire by themselves; and 2. pregnant women who refused to participate in the study. The sample size calculation formula for cross-sectional studies was used to calculate the minimum theoretical sample size for this study. According to the prevalence of DV which have been reported in a previous study [29], admissible error was 0.1, α = 0.05. 763 people were required in order for the participants to represent the population. There were 819 registered samples in the 14 selected communities, of which 6 were excluded because of refusals to respond and failure to contact; therefore, 813 pregnant women who met the requirements participated in the study. The response rate was 99.3% (813/819).

All subjects gave their informed consent for inclusion before they participated in the study. The study was conducted in accordance with the Declaration of Helsinki, and the protocol was approved by the Ethics Committee of Xiangya School of Public Health, Central South University (XYGW-2019-056).

### Data collection and measurements

#### General characteristics

General characteristics were collected, including age, ethnicity, marital status, monthly per capita household income, education level, occupation, partner’s education level, partner’s occupation, whether they had medical insurance, whether they had a smoking habit, whether they had a drinking habit, the partner’s smoking habit, the partner’s drinking habit, the mode of pregnancy, history of delivery, pregnancy intention, whether they had experienced multiple abortions/sterility, whether they had received antenatal examinations, whether they had pregestational diseases (depression or diabetes), and whether they had pregnancy complications (gestational diabetes, pregnancy-induced hypertension, intrahepatic cholestasis of pregnancy, and others). Being married was defined as being in a stable marriage. Unstable marriages included unmarried, divorced, and widowed.

#### Family factors

Family factors consisted of four aspects: family relationships, family structure, family-related stressors and family function. Family relationships were evaluated on two dimensions: the relations with the mother-in-law and relations with other family members. Good or bad relationships with family members were judged subjectively by the participants. Family structure included the number of cohabitants and whether they lived with elders. In terms of family-related stressors, three family negative life events were listed: whether they had medium household debt, whether they were separated from their partner, and whether their partner had extramarital affairs. The Family Adaptation Partnership Growth Affection and Resolve Index (APGARI) is a tool for evaluating family functions. The Family APGARI has five items, and each item was answered on a 3-point Likert scale from “often” (2 points) to “rarely” (0 points). The total score was 0–10 points. High family APGARI scores ranging from 7 to 10 points indicated good family function, middle family APGARI scores ranging from 4 to 6 points indicated moderate family dysfunction, and low family APGARI ranges from 0 to 3 points indicated severe family dysfunction. Family APGARI has been widely used and has good reliability and validity [30, 31]. The Cronbach’s α is 0.876.

#### Assessment tools for DV

The Abuse Assessment Screen Questionnaire (AAS) was compiled in 1995 by McFarlane and translated into Chinese by Leung of the University of Hong Kong and was used to assess DV during pregnancy. There are eight items in this scale assessing mental, physical and sexual violence as well as the psychological response to perpetrators in three periods: lifetime and 12 months prior to and during pregnancy. The scale provides participants several common perpetrators to choose from (husband, ex-husband, boyfriend, stranger and others). The response to each item was either Yes or No. If the interviewee answered “Yes” to one or more of Questions 5 to 7, she was identified as a victim of DV during pregnancy [32, 33]. The scale’s Cronbach’s α is 0.685. The retest reliability of the scale was 0.91, the specificity was 97–99%, and the sensitivity to mild and severe violence was 32% and 61-94%, respectively [33, 34]. These scales are widely known as self-management tools for screening DV against pregnant women with good validity and reliability [27].

### Statistical analysis

*EpiData 3.1* and *SPSS 19.0* software were used for data entry and statistical analysis. Categorical variables are expressed as *n (%)*, the χ2 test was applied for comparisons of general and family characteristics between women who had experienced DV and who had not (no DV group) during the pregnancy. The crude odds ratio (COR) was reported by multivariate binary logistic regression models. For instance, some general characteristics will be adjusted, and adjusted odds ratio (AOR) and 95% confidence interval (95% CI) were reported. The statistical significance level was accepted as p<0.05. All statistical tests were 2-sided.

## Results

The mean age of the 813 participants was 28.98 ± 4.52 years. Most of them were ethnic Han (*n* = 798, 98.2%), had stable marital status (*n* = 728, 89.5%) and were employed (*n* = 599, 73.7%). More than half of them had college / university degree or above education level (*n* = 472, 58.1%). A total of 9% had a monthly household income of ≤3000, 36.9% of 3001–5000, 33.5% of 5001–8000, and 20.7% of > 8001. The majority had medical insurance (*n* = 592,72.8%). 3 (0.4%) and 80 (9.8%) had smoking and drinking habits, respectively. Regarding pregnant women’s partner, 487 (59.9%) had college / university degree or above education level, and 799 (98.3%) were employed. A total of 288 (35.4%) and 314 (38.6%) had smoking and drinking habits, respectively. (Table 2).

During pregnancy, 127 (15.62%) participants suffered from DV in at least one form. Mental violence was the most serious (*n* = 90, 11.07%), followed by physical and sexual violence, which only occurred in 8 (0.98%) and 7(0.86%), respectively. A total of 25 (3.08%) participants reported that they had experienced two types of violence (physical and mental violence), and 44 (5.41%) participants reported that they were afraid of the person who hurt them. (Table 1).

**Table 1:** Distribution of different types of domestic violence (*n* = 813)

The type of DV	n (%)
Only physical violence	8 (0.98)
Only mental violence	90(11.07)
Only sexual violence	7 (0.86)
Double violence (both physical and mental violence)	25(3.08)

### Differences in general characteristics and family factors were compared between the two groups

According to Tables 2 and 3, nine variables showed statistically significant differences between the DV group and the no DV group (p<0.05): whether they had medical insurance, whether they had a drinking habit, whether they had experienced multiple abortions/sterility, the relations with the mother-in-law, relations with other family members, whether they had medium household debt, whether they were separated from their partner, and whether their partner had extramarital affairs.

**Table 2:** General characteristics of the study sample in this study. (DV vs No DV)

Variables	DV (*n* = 127)	No DV (*n* = 686)	Total (*n* = 813)	χ^2^ value	*p* value
Age				0.189	0.910
<26	35 (27.6)	202 (29.4)	237 (29.2)		
27–32	67 (52.8)	354 (51.6)	421 (51.8)		
>33	25 (19.7)	130 (19.0)	155 (19.1)		
Ethnicity				0.690	0.406
Minority	4 (3.1)	11 (1.6)	15 (1.8)		
Han ethnic	123 (96.9)	675 (98.4)	798 (98.2)		
Marital status				0.739	0.390
Stable	111 (87.4)	617 (89.9)	728 (89.5)		
Unstable	16 (12.6)	69 (10.1)	85 (10.5)		
Education				1.064	0.302
Senior school / technical school or less	48 (37.8)	293 (42.7)	341 (41.9)		
College / university degree or above	79 (62.2)	393 (57.3)	472 (58.1)		
Occupation				0.944	0.331
Employed	98 (77.2)	501 (73)	599 (73.7)		
Unemployed	29 (22.8)	185 (27)	214 (26.3)		
Education (partner)				1.064	0.302
Senior school / technical school or less	48 (37.8)	293 (42.7)	341 (41.9)		
College / university degree or above	79 (62.2)	393 (57.3)	472 (58.1)		
Occupation (partner)				0.019	0.890
Employed	125 (98.4)	674 (98.3)	799 (98.3)		
Unemployed	2 (1.6)	12 (1.7)	14 (1.7)		
Monthly per capita household income				4.025	0.259
≤ 3000	12 (9.4)	61 (8.9)	73 (9.0)		
3001–5000	44 (34.6)	256 (37.3)	300 (36.9)		
5001–8000	51 (40.2)	221 (32.2)	272 (33.5)		
>8001	20 (15.7)	148 (21.6)	168 (20.7)		
Medical insurance				**4.275**	**0.039**
Yes	102 (80.3)	490 (71.4)	592 (72.8)		
No	25 (19.7)	196 (28.6)	221 (27.2)		
Smoking habit				0.002	0.960
Yes	1 (0.8)	2 (0.3)	3 (0.4)		
No	126 (99.2)	684 (99.7)	810 (99.6)		
Drinking habit				**9.499**	**0.002**
Yes	22 (17.3)	58 (8.5)	80 (9.8)		
No	105 (82.7)	628 (91.5)	733 (90.2)		
Smoking habit (partner)				0.042	0.838
Yes	46 (36.2)	242 (35.3)	288 (35.4)		
No	81 (63.8)	444 (64.7)	525 (64.6)		
Drinking habit (partner)				1.393	0.238
Yes	55 (43.3)	259 (37.8)	314 (38.6)		
No	72 (56.7)	427 (62.2)	499 (61.4)		
Mode of pregnancy				1.113	0.291
Natural conception	124 (97.6)	656 (95.6)	780 (95.9)		
Artificial impregnation	3 (2.4)	30 (4.4)	33 (4.1)		
History of delivery				0.388	0.533
≤ 1	66 (89.2)	347 (86.5)	413 (86.9)		
>1	8 (10.8)	54 (13.5)	62 (13.1)		
Pregnancy intention				0.958	0.328
Intended	70 (55.1)	410 (59.8)	480 (59.0)		
Unintended	57 (44.9)	276 (40.2)	333 (41.0)		
Multiple abortions/sterility				**11.476**	**0.001**
Yes	16 (12.6)	33 (4.8)	49 (6.0)		
No	111 (874)	653 (95.2)	764 (94.0)		
Regular antenatal examination				1.836	0.175
Yes	120 (94.5)	623 (90.8)	743 (91.4)		
No	7 (5.5)	63 (9.2)	70 (8.6)		
Pregestational diseases				0.676	0.411
Yes	3 (2.4)	7 (1.0)	10 (1.2)		
No	124 (97.6)	679 (90.0)	803 (98.8)		
Pregnancy complications				3.264	0.071
Yes	19 (15.0)	66 (9.6)	85 (10.5)		
No	108 (85.0)	620 (90.4)	728 (89.5)		

Abbreviations: *DV* domestic violence

Data are presented as n (%)

Characters in bold means statistical significance *p*<0.05

**Table 3:** Family characteristics of the study sample in this study. **(DV vs No DV)**

Variables	DV (*n* = 127)	No DV (*n* = 686)	Total(*n* = 813)	χ^2^ value	*p* value
Family relationships					
Relationship with mother-in-law				**45.411**	**0.000**
Good	106 (83.5)	668 (97.4)	774		
Bad	21 (16.5)	18 (2.6)	39		
Relationship with other families				**49.391**	**0.000**
Good	104 (81.9)	666 (97.1)	770		
Bad	23 (18.1)	20 (2.9)	43		
Family structure					
Number of cohabitation (include oneself)				0.058	0.809
1–2	26 (20.5)	147 (21.4)	173		
>3	101 (79.5)	539 (78.6)	640		
Live with eldership				0.813	0.367
Yes	88 (69.3)	447 (65.2)	535		
No	39 (30.7)	239 (34.8)	278		
Family related stressors					
Medium household debt				**8.857**	**0.003**
Yes	20 (15.7)	52 (7.6)	72		
No	107 (84.3)	624 (92.4)	741		
Separation				**8.095**	**0.004**
Yes	29 (22.8)	90 (13.1)	119		
No	98 (77.2)	596 (85.9)	694		
Partner’s extramarital affairs				**18.347**	**0.000**
Yes	9 (7.1)	8 (1.2)	17		
No	118 (92.9)	678 (98.8)	796		
Family function					
Family APGAR index				**40.096**	**0.000**
Low level	25 (19.7)	41 (6)	66		
Middle level	52 (40.9)	204 (29.7)	256		
High level	50 (39.4)	441 (64.3)	491		

Abbreviations: *DV* domestic violence

Data are presented as n (%)

Characters in bold indicate statistical significance *p*<0.05

### The result of multivariate binary logistic regression analysis

These six variables were adjusted as confounding variables (medical insurance, drinking habit, multiple abortions/sterility, age, ethnicity, marital status). After adjustment, the results of multivariate binary logistic regression showed that the risk factors for DV were tensions with their mother-in-law (OR: 2.85; 95% CI: 1.29 to 6.30), tensions with other family members (OR: 3.30; 95% CI: 1.57 to 6.93), medium household debt (OR: 2.17; 95% CI: 1.18 to 4.00), middle and low family APGARI (OR: 2.01; 95% CI: 1.30 to 3.13, OR: 4.01; 95% CI: 2.09 to 7.69) (Table 4).

**Table 4:** Multivariate binary logistic regression analysis of family factors associated with DV among pregnant women

Variables	COR^a^ (95% CI)	AOR^b^ (95% CI)
Family relationships		
Relationship with mother-in-law		
Good	**1.00**	**1.00**
Bad	**2.71 (1.24, 5.90)**	**2.85 (1.29, 6.30)**
Relationship with other families		
Good	**1.00**	**1.00**
Bad	**3.40(1.63,7.09)**	**3.30 (1.57, 6.93)**
Family structure		
Number of cohabitation (include oneself)		
1–2	1.34 (0.71, 2.51)	1.00
>3	1.00	1.19 (0.63, 2.26)
Live with eldership		
Yes	1.29 (0.83, 2.00)	1.30 (0.83, 2.03)
No	1.00	1.00
Family related stressors		
Medium Household debt		
Yes	**2.03 (1.10, 3.74)**	**2.17 (1.18, 4.00)**
No	**1.00**	**1.00**
Separation		
Yes	1.55 (0.93, 2.60)	1.61 (0.96, 2.70)
No	1.00	1.00
Partner’s extramarital affairs		
Yes	**3.06 (1.01, 9.30)**	2.37 (0.74, 7.54)
No	**1.00**	1.00
Family function		
Family APGAR index		
Low level	**3.52 (1.85, 6.69)**	**4.01 (2.09, 7.69)**
Middle level	**1.94 (1.25, 3.01)**	**2.01 (1.30, 3.13)**
High level	**1.00**	**1.00**

Abbreviations: *DV* domestic violence, *COR* crude odds ratio, *AOR* adjusted odds ratio

^a^ Multivariate binary logistic regression model

^b^Some general characteristics were adjusted (medical insurance, drinking habit, multiple abortions/sterility, age, ethnicity, marital status)

Characters in bold indicate statistical significance, *p*<0.05

## Discussion

In this study, 127 pregnant women were identified as victims of DV (15.62%). Of these participants, 90 experienced mental violence (11.07%), 8 experienced physical violence (0.98%), 7 experienced sexual violence (0.86%), and 25 reported experiencing two forms of violence (3.08%). The prevalence of DV in this study was similar to that of another study carried out in Changsha City, Hunan Provinces, China (the positive rate was 11.3%) [1]. However, the prevalence in this study was lower than those in some other countries. In a Portuguese study, 43.4% reported a history of DV during pregnancy [35]. A hospital-based cross-sectional study carried out in Northwest Ethiopia reported that 58.7% were victims of DV during pregnancy [24]. The possible reasons for the lower positive rate in this study may be that people from different regions and cultural backgrounds have different understandings of DV, leading the fact that some women who are suffering from violence do not regard these behaviours as DV [27]. In Eastern culture, women were more likely to be silent to maintain the stability of the family and to avoid more serious violence, which might have led to a decline in the positive reporting rate [36]. Furthermore, the outcome assessment tool might the possible reason for the differences in the prevalence of DV. The study conducted in Changsha used the Abuse Assessment Screen (AAS), whereas the studies in Portugal and Ethiopia used the WHO tool to assess the outcome variable. Comparing these two scales, we can find that the WHO tool is more specific and detailed in describing violent behaviours, such as occasional verbal and physical conflicts, which are easy to be associated with and identified as domestic violence, thus showing a higher positive rate. In terms of the types of DV, mental violence was the most serious, which was similar to findings in Guatemala and Iran [37, 38]. The possible reason for this phenomenon is that physical and sexual violence was often accompanied by mental violence, and mental violence almost always precedes physical violence, or could be independent of physical and sexual violence [39, 40]. Most of the perpetrators are the intimate partner of pregnant women (husband, ex-husband, boyfriend), which was consistent with the study by Mtonga in Zambia [41]. This study did not explore the characteristics of different perpetrator, and further research in this aspect would be needed.

The findings showed that tensions with family members, especially the mother-in-law, was significantly related to the occurrence of DV during pregnancy (p<0.05). The risk of DV was 1.85 times higher in pregnant women who have strained relation with their mother-in-law than that in good relation group (OR:2.85; 95%CI: 1.29 to 6.30), these findings were almost consistent with those reported by Chen in Hong Kong, China [42]. In China, discord between family members, especially contradictions between mother-in-law and daughter-in-law, are important factors of family conflict. Due to the differences in fertility and health concepts between women and their mothers-in-law [43], contradictions occur frequently, especially during pregnancy. On the other hand, it is also possible that after the conflict between husband and wife, it is common for a mother-in-law to follow the will of her son by disciplining a wife, resulting in tensions between mother-in-law and daughter-in-law [42].

In this study, medium household debt were associated with the occurrence of DV (p<0.05). The risk in participants who had medium household debt was 2.17 times as much as that in the reference group (OR: 2.17; 95% CI: 1.18 to 4.00). Thus, pregnant women with medium household debt were more likely to suffer from DV. This was consistent with results from the research by Tsui in Hong Kong [44]. During pregnancy, with the increasing household expenses accompanied by the loss of working ability of the pregnant woman, pregnant women will more dependent on their husbands in economic, which will easily leads to disagreement and violence [24].

Although the differences in extramarital affairs between the two groups were not statistically significant after adjustment, we cannot ignore the possible association between this factor and the occurrence of DV. Because this association has been approved by Peedicayil et al. [45]. The extramarital affairs of women’s partner often occurred during the pregnancy [46]. During pregnancy, women paid more attention to their own physiological changes and the foetal growth, and they sometimes ignore the emotional and sexual needs of their male partners, which can cause family conflicts and violence to some extent [47].

The results of this study suggest that family dysfunction was a risk factor for DV, this finding was similar to the study by Tuesca in Colombia [48]. The lower the family APGARI indicates the more serious the family dysfunction. The risk for participants who with low and middle family APGARI was 4 and 2 times as much as that of pregnant women with a high family APGARI (OR: 4.01; 95% CI: 2.09 to 7.69; OR: 2.01; 95% CI: 1.30 to 3.13), respectively. Family dysfunction reflected that pregnant women could not acquire enough care, love and help from their families and even that the family might be the source of bodily injury and mental pressure.

In this study, the selection of the sample is representative. The principle of randomization was followed, and women in the third trimester of pregnancy were selected as the subjects of the survey. Women in the third trimester of pregnancy have experienced nearly the entire pregnancy process and can report fully on their experiences of abuse throughout pregnancy with less recall bias. Equally important, pregnant women have different mental activities and physiological characteristics at different stages of pregnancy. Compared with all periods of pregnant women, the choice of women in the third trimester can reduce the influence of confounding factors such as gestational age. This study provides a comprehensive discussion of the relationship between family factors and pregnant women’s DV from various dimensions, which has certain innovative value in the prevention of DV. The present study also had certain limitations. This study was cross-sectional, thus, there were limitations in identifying causal relationships. Second, because of the self-reported design, we cannot deny the reporting bias. Last, there are some limitations to the AAS scale: AAS cannot distinguish the severity of DV and injured body parts, which may reduce the investigative effect of the scale, so that some risk characteristics cannot be identified. However, this study promoted an understanding of the relationship between family factors and DV and further confirmed the importance of family factors.

## Conclusions

This study reported 15.62% positive rates of DV among women in the third trimester of pregnancy in Hengyang City, China. Of the three major forms of DV, mental violence has the highest incidence (11.07%), and 3.08% of participants reported that they had experienced two types of violence. There were strong associations between DV and family tensions, medium household debt, and family dysfunction among women in late pregnancy. The issue of DV in pregnancy has been overlooked in a long time, maternal and child health personnel should pay more attention to identifying pregnant women with these potential risk family characteristics. And we recommend domestic violence screening was set as a part of prenatal care for pregnant women so that relevant departments can provide appropriate and timely law enforcement, safe shelter and legal aid to decrease damage and even avoid the occurrence of DV. At the same time, just as Cripe et al. reported that asking pregnant women about violence may be an intervention, because women’s recognition and disclosure of abusive behaviour will make them realize that abusive behaviour is common and their feelings are valued [29]. So, we should focus on women and empower them to help them strengthen the sense of self-protection, face their own value and gain better control of their lives. These findings have positive significance for the screening and prevention of DV in pregnant women.

## Data Availability

Data is currently not available online. But can be made available to any interested person(s) contacting the corresponding author via email.

## References

[1] Zhang Y, Zou SH, Cao YP, Zhang YL (2012). Relationship between domestic violence and postnatal depression among pregnant Chinese women. Int J Gynecol Obstet.

[2] Sarkar NN (2008). The impact of intimate partner violence on women's reproductive health and pregnancy outcome. J Obstetrics Gynaecol.

[3] Garcia-Moreno C, Pallitto C, Devries K, Stockl H, Watts C, Abrahams N (2013). Global and regional estimates of violence against women: prevalence and health effects of intimate partner violence and non-partner sexual violence.

[4] Mavrikiou PM, Apostolidou M, Parlalis SKJSSJ (2014). Risk factors for the prevalence of domestic violence against women in Cyprus. Soc Sci J.

[5] Goodwin MM, Gazmararian JA, Johnson CH, Gilbert BC, Saltzman LE (2000). Pregnancy Intendedness and physical abuse around the time of pregnancy: findings from the pregnancy risk assessment monitoring system, 1996–1997. Maternal Child Health J.

[6] Gelles RJ (1988). Violence and pregnancy: are pregnant women at greater risk of abuse?. J Marriage Fam.

[7] Brownridge DA, Taillieu TL, Tyler KA, Tiwari A, Chan KL, Santos S (2011). Pregnancy and intimate partner violence: risk factors, severity, and health effects. Violence Against Women.

[8] Jasinski JL (2004). Pregnancy and domestic violence: a review of the literature. Trauma, Violence, & Abuse.

[9] Laude M, Fuchs SC, Stallings RY, Patricia P, Christine K, Josephine R, Campbell DW, Jacquelyn C, Sara T. Abuse During and Before Pregnancy: Prevalence and Cultural Correlates. Violence Vict. 2000;15(3):303–21. 11200104

[10] Jahanfar S, Malekzadegan Z (2007). The prevalence of domestic violence among pregnant women who were attended in Iran University of Medical Science Hospitals. J Fam Violence.

[11] Johnson JK, Haider F, Ellis K, Hay DM, Lindow SW (2003). The prevalence of domestic violence in pregnant women. BJOG.

[12] Abadi MNL, Ghazinour M, Nojomi M, Richter J (2012). The buffering effect of social support between domestic violence and self-esteem in pregnant women in Tehran. Iran J Family Violence.

[13] Krmaliani R, Ifan F, Bann CM, Mcclure EM, Moss N, Pasha O, Goldenberg RL (2008). Domestic violence prior to and during pregnancy among Pakistani women. Acta Obstet Gynecol.

[14] Perales MT, Cripe SM, Lam N, Sanchez SE, Sanchez E, Williams MA (2009). Prevalence, types, and pattern of intimate partner violence among pregnant women in Lima, Peru. Violence against women.

[15] Makayoto LA, Omolo J, Kamweya AM, Harder VS, Mutai J (2013). Prevalence and associated factors of intimate partner violence among pregnant women attending Kisumu District hospital, Kenya. Matern Child Health J.

[16] Connolly AM, Katz V, Bash K, McMahon M, Hansen W (1997). Trauma and pregnancy. Am J Perinatol.

[17] Newberger EH, Barkan SE, Lieberman ES, Mccormick MC, Schechter SJJ (1992). Abuse of pregnant women and adverse birth OutcomeCurrent knowledge and implications for practice. JAMA.

[18] Petersen R, Gazmararian JA, Spitz AM, Rowley DL, Marks JS (1997). Violence and adverse pregnancy outcomes: a review of the literature and directions for future research. Am J Prev Med.

[19] Gazmararian JA, Lazorick S, Spitz AM, Ballard TJ, Saltzman LE, Marks JS (1996). Prevalence of violence against pregnant women. JAMA.

[20] Taillieu TL, Brownridge DA (2010). Violence against pregnant women: prevalence, patterns, risk factors, theories, and directions for future research. Aggression Violent Behavior.

[21] Saltzman LE, Johnson CH, Gilbert BC, Goodwin MM (2003). Physical abuse around the time of pregnancy: an examination of prevalence and risk factors in 16 states. Matern Child Health J.

[22] Covington DL, Hage M, Hall T, Mathis M (2001). Preterm delivery and the severity of violence during pregnancy. J Reprod Med.

[23] Dunn LL, Oths KS (2004). Prenatal predictors of intimate partner abuse. J Obstet Gynecol Neonatal Nurs.

[24] Fekadu E, Getachew Y, Alemu GK, Awoke AT, Tameru M, Tinsae G, Fetene TD (2018). Prevalence of domestic violence and associated factors among pregnant women attending antenatal care service at University of Gondar Referral Hospital. Northwest Ethiopia BMC Women's Health.

[25] Heaman MI, Gupton AL, Moffatt ME (2005). Prevalence and predictors of inadequate PrenatalCare: a comparison of aboriginal andNon-aboriginal women in Manitoba. Journal of Obstetrics & Gynaecology Canada.

[26] Hellmuth JC, Gordon KC, Stuart GL, Moore TM (2013). Risk factors for intimate partner violence during pregnancy and postpartum. Archives of Womens Mental Health.

[27] Leung WC, Leung TW, Lam YY, Ho PC (1999). The prevalence of domestic violence against pregnant women in a Chinese community. Int J Gynaecol Obstet.

[28] Martin SL, Curtis S (2004). Gender-based violence and HIV/AIDS: Recognising links and acting on evidence. Lancet.

[29] Cripe SM, Sanchez SE, Sanchez E, Quintanilla BA (2010). Intimate partner violence during pregnancy: a pilot intervention program in Lima. Peru J Interpersonal Violence.

[30] Smilkstein G, Ashworth C, Montano D (1982). Validity and reliability of the family APGAR as a test of family function. J Family Pract.

[31] Liu Y, Duan XL, Li Z (2012). Influence of social support and family function on the approach of delivery. J Modern Clin Med.

[32] Rabin RF, Jennings JM, Campbell JC, Bair-Merritt MH (2009). Intimate Partner Violence Screening Tools: A Systematic Review.

[33] Reichenheim ME, Moraes CL (2004). Comparison between the abuse assessment screen and the revised conflict tactics scales for measuring physical violence during pregnancy. J Epidemiol Community Health.

[34] Rabin RF, Jennings JM, Campbell JC (2009). Intimate partner violence screening tools: a systematic review. Am J Prev Med.

[35] Almeida FSJ, Coutinho EC, Duarte JC, Chaves CMB, Nelas PAB, Amaral OP, Parreira VC (2017). Domestic violence in pregnancy: prevalence and characteristics of the pregnant woman. J Clin Nurs.

[36] Tang CS, Wong D, Cheung FM (2002). Social construction of women as legitimate victims of violence in Chinese societies. Violence Against Women.

[37] Mohamadian F, Hashemian A, Bagheri M, Direkvand-Moghadam A (2016). Prevalence and risk factors of domestic violence against Iranian women: a cross-sectional study. Korean Journal of Family Medicine.

[38] Johri M, Morales RE, Boivin JF, Samayoa BE, Hoch JS, Grazioso CF, Barrios Matta IJ, Sommen C, Baide Diaz EL, Fong HR (2011). Increased risk of miscarriage among women experiencing physical or sexual intimate partner violence during pregnancy in Guatemala City, Guatemala: cross-sectional study. Bmc Pregnancy Childbirth.

[39] O’Leary K (1999). Psychological abuse: a variable deserving critical attention in domestic violence. Violence Vict.

[40] Hou J, Yu L, Ting SMR (2011). The status and characteristics of couple violence in China. J Fam Violence.

[41] Mtonga MDM (2010). The prevalence and factors contributing to domestic violence among pregnant women attending antenatal clinics in Lusaka urban clinics in Zambia. Injury Prevention.

[42] Chan KL, Brownridge DA, Tiwari A, Fong DYT, Leung WC (2008). Understanding violence against Chinese women in Hong Kong: an analysis of risk factors with a special emphasis on the role of in-law conflict. Violence Against Women.

[43] Turner MJ, Young CR, Black KI (2006). Daughters-in-law and mothers-in-law seeking their place within the family: a qualitative study of differing viewpoints. Fam Relat.

[44] Tsui KL, Chan AY, So FL, Kam CW (2006). Risk factors for injury to married women from domestic violence in Hong Kong. Hong Kong Med J.

[45] Peedicayil A, Sadowski LS, Jeyaseelan L (2004). Spousal physical violence against women during pregnancy. BJOG Int J Obstet Gynaecol.

[46] Onah HE, Iloabachie GC, Obi SN, Ezugwu FO, Eze JN (2002). Nigerian male sexual activity during pregnancy. Int J Gynecol Obstet.

[47] Feder G, Davies RA, Baird K (2011). Identification and referral to improve safety (IRIS) of women experiencing domestic violence with a primary care training and support programme: a cluster randomised controlled trial. Lancet.

[48] Tuesca R, Borda M (2003). Violencia física marital en Barranquilla (Colombia): prevalencia y factores de riesgo. Gac Sanit.

